# Comprehensive DNA Methylation Analysis of Human Neuroblastoma Cells Treated With Haloperidol and Risperidone

**DOI:** 10.3389/fnmol.2021.792874

**Published:** 2021-12-06

**Authors:** Jianbin Du, Yutaka Nakachi, Tomoki Kiyono, Shinya Fujii, Kiyoto Kasai, Miki Bundo, Kazuya Iwamoto

**Affiliations:** ^1^Department of Molecular Brain Science, Graduate School of Medical Sciences, Kumamoto University, Kumamoto, Japan; ^2^Department of Neuropsychiatry, Graduate School of Medicine, The University of Tokyo, Tokyo, Japan; ^3^The International Research Center for Neurointelligence (WPI-IRCN), The University of Tokyo Institutes for Advanced Study (UTIAS), Tokyo, Japan; ^4^University of Tokyo Center for Integrative Science of Human Behavior (CiSHuB), Tokyo, Japan

**Keywords:** schizophrenia, DNA methylation, haloperidol, risperidone, epigenetics, neuroblastoma

## Abstract

Accumulating evidence suggests that the epigenetic alterations induced by antipsychotics contribute to the therapeutic efficacy. However, global and site-specific epigenetic changes by antipsychotics and those shared by different classes of antipsychotics remain poorly understood. We conducted a comprehensive DNA methylation analysis of human neuroblastoma cells cultured with antipsychotics. The cells were cultured with low and high concentrations of haloperidol or risperidone for 8 days. DNA methylation assay was performed with the Illumina HumanMethylation450 BeadChip. We found that both haloperidol and risperidone tended to cause hypermethylation changes and showed similar DNA methylation changes closely related to neuronal functions. A total of 294 differentially methylated probes (DMPs), including 197 hypermethylated and 97 hypomethylated DMPs, were identified with both haloperidol and risperidone treatment. Gene ontology analysis of the hypermethylated probe-associated genes showed enrichment of genes related to the regulation of neurotransmitter receptor activity and lipoprotein lipase activity. Pathway analysis identified that among the DMP-associated genes, *SHANK1* and *SHANK2* were the major genes in the neuropsychiatric disorder-related pathways. Our data would be valuable for understanding the mechanisms of action of antipsychotics from an epigenetic viewpoint.

## Introduction

Schizophrenia is a major psychiatric disorder characterized by positive and negative symptoms as well as cognitive impairments. Schizophrenia affects approximately 1% of the population and has become a worldwide public health concern with a considerable financial burden on individuals, families and society (Insel, 2010). Although genetic factors are involved in the etiology of schizophrenia, genes with large effect sizes have not been identified. In addition to the complex gene–environment interactions, recent genetic studies suggest that multiple genetic factors with small effect sizes are involved in its etiology (Purcell et al., 2009, 2014; Schizophrenia Working Group of the Psychiatric Genomics Consortium, 2014).

Epigenetics is the study of heritable and stable changes in gene expression that are not caused by changes in DNA sequences, and it involves DNA methylation and histone modifications as the major molecular mechanisms (Bird, 2002). Altered epigenetic status has been proposed to be involved in the pathophysiology of major psychiatric disorders, including schizophrenia (Nishioka et al., 2012; Nestler et al., 2016; Richetto and Meyer, 2020; Bundo et al., 2021; Ueda et al., 2021).

In addition, accumulating evidence also suggests that antipsychotics affect epigenetic status in the brain (Kurita et al., 2013; Swathy and Banerjee, 2017; de la Fuente Revenga et al., 2019). However, previous studies have mainly examined their effects on histone modification activities, and the global and site-specific effects of antipsychotics on DNA methylation are poorly understood.

We previously performed comprehensive DNA methylation analyses of the antipsychotics blonanserin and perospirone using human neuroblastoma cells (Murata et al., 2014, 2019). Blonanserin and perospirone are classified as atypical antipsychotics that block both dopaminergic and serotonergic signaling pathways. Both antipsychotics generally increase the DNA methylation levels of CpG sites far from the promoter regions in a dose-dependent manner and affect the epigenetic status of their pharmacological targets, such as dopamine receptor 2 (*DRD2*) and serotonin receptor 2A (*HTR2A*).

To further understand the epigenetic effects of antipsychotics, we conducted a comprehensive DNA methylation analysis of haloperidol, a typical antipsychotic, and risperidone, an atypical antipsychotic, using the same cell culture model with a HumanMethylation450 BeadChip.

## Materials and Methods

### Cell Culture

Human neuroblastoma SK-N-SH cells (American Type Culture Collection, Manassas, VA, United States) were cultured as previously described (Asai et al., 2013; Sugawara et al., 2015). In brief, the cells were cultured with Eagle’s minimal essential medium with 10% fetal bovine serum (FBS) containing antipsychotics for 8 days. In this study, we used low and high concentrations of haloperidol (1 and 10 μM; Sigma-Aldrich, St Louis, MO, United States) and risperidone (3 and 30 μM; Sigma-Aldrich) based on their effective blood concentrations. The antipsychotics were dissolved in dimethyl sulfoxide (DMSO). Final concentrations of DMSO were 0.001–0.06%, depending on the dose of antipsychotics. The cells were cultured with or without 0.06% of DMSO for 8 days as a control. Three independent samples were prepared for each experimental group (*n* = 18 in total).

### DNA Methylation Assay

From each sample, genomic DNA was extracted via standard phenol:chloroform methods. The DNA samples were subjected to bisulfite modification by the EZ DNA Methylation Kit D500 (Zymo Research, Irvine, CA, United States). A comprehensive DNA methylation assay was performed with the HumanMethylation450 BeadChip (Illumina, Inc., San Diego, CA, United States) according to the manufacturer’s instructions. The DNA methylation level of each probe was represented by a β value. It was calculated according to the ratio of intensities between methylated and unmethylated alleles, as in the following formula: β value = methylated intensity/(methylated intensity + unmethylated intensity + 100).

### Data Analysis

Among the 485,512 probes on the chip, we excluded unreliable probes whose detection p values ≥0.01 in at least one experimental sample. The remaining data were analyzed using ChAMP (Tian et al., 2017) and minfi (Aryee et al., 2014) packages in R software (version 4.0.2).^1^ Probes for CpG sites on the sex chromosomes or associated with single nucleotide polymorphisms and those for non-CpG sites were removed, resulting in 411,750 probes for further analysis. Data analysis including annotation was based on the HumanMethylation450 v1.2 Manifest provided from the manufacture (Illumina). Principal component analysis (PCA) was performed using the FactoMineR (Lê et al., 2008) and factoextra^2^ packages in R. Unsupervised hierarchical clustering was performed on probes using the Euclidean distance metric and complete linkage methods using the stats package^1^. Heatmap visualizations were created using pheatmap (Kolde, 2019). Probes that showed Benjamini-Hochberg (BH) adjusted *P*-value <0.05 in moderated t test in ChAMP and that absolute difference in β value (|Δβ|) ≥ 0.1 were considered to have significant methylation alterations. Gene Ontology (GO) term enrichment analysis and visualization were performed using Cytoscape v3.6.1 (Shannon et al., 2003) and the ClueGO plugin v2.5.7 (Bindea et al., 2009) with a two-sided hypergeometric test, Bonferroni step-down corrected p-value cutoff = 0.05, medium specificity, and a Kappa score of >0.4. The Venn diagram was created using the Draw Venn Diagram tool.^3^

## Results

### Cluster Analysis and Principal Component Analysis

**FIGURE 1:**
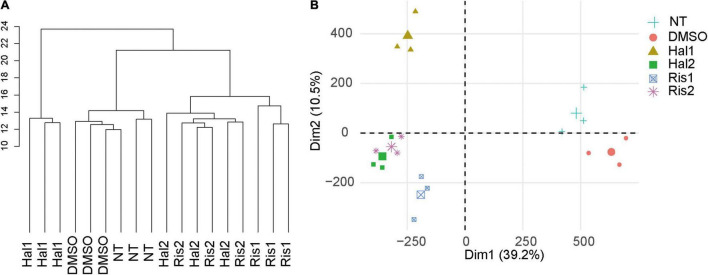
Cluster analysis and PCA. **(A)** Unsupervised hierarchical cluster analysis of all samples. Scale indicates Euclidean distance. **(B)** PCA of all samples. The large symbol indicates the average value of three experiments. NT, no treatment control group; DMSO, dimethyl sulfoxide control group; Hal1, high dose haloperidol group; Hal2, low dose haloperidol group; Ris1: high dose risperidone group, Ris2: low dose risperidone group. PCA, principal component analysis.

We cultured human neuroblastoma SK-N-SH cells with two doses of haloperidol or risperidone for 8 days. Doses were determined based on their minimum and maximum effective blood concentrations. After culture, we obtained DNA methylation profiles with the Illumina HumanMethylation450K BeadChip. After removing the unreliable probes, we obtained 411,750 probes for data analysis. Unsupervised hierarchical clustering of all β values, which are proxies of DNA methylation levels, showed that the antipsychotic groups and the two control groups, including the no treatment (NT) and DMSO group, were separated (Figure 1A). PCA also revealed the separation of the antipsychotic and control groups (Figure 1B). We examined the correlation of β values between the NT and DMSO groups and found a strong correlation (R = 0.999, Supplementary Figure 1). Based on this result, we concluded that DMSO has a negligible effect on the DNA methylation status of cell culture in our study. We compared the β values of the antipsychotic groups with that of the DMSO group and defined differentially methylated probes (DMPs) as probes showing a BH-adjusted *P*-value below 0.05 in the moderated t test in ChAMP (Tian et al., 2017) and an absolute β value difference ≥0.1. We first examined the genomic distribution and direction of changes of DMPs in each dose of antipsychotics and then examined the characteristics of DMPs between two doses. Finally, we examined the characteristics of common DMPs between the two antipsychotics.

### Comprehensive DNA Methylation Analysis of Haloperidol

We identified 3,028 and 1,320 DMPs in the high and low concentration groups for haloperidol, respectively. The average β values of DMPs in the high-dose group was significantly increased compared to those in the control group (Table 1). Probes located much farther from the promoter regions showed significant increases in β values, including the 5′-UTR, gene body and intergenic regions. In the low-dose group, although there was no significant alteration in the β value among all DMPs, DMPs in the gene body showed a significant increase similar to the high-dose group (Table 1). Concordantly, 72% of DMPs showed an increase in the DNA methylation level in the high-dose group, and this rate was only 55% in the low-dose group (Supplementary Figure 2). However, 76 and 69% of DMPs showed increased methylation in the high- and low-dose groups, respectively, when looking at the gene body. With regard to the context of CpG islands, hypermethylated probes were enriched in the shelves and open seas at both doses (Table 1 and Supplementary Figure 2).

**TABLE 1:** Average β values of DMPs in the haloperidol treatment group according to the genomic positions.

Dose	Context	# of probes	β value (haloperidol)*	β value (control)*	*P* value
High	All DMPs	3028	0.520 ± 0.189	0.468 ± 0.169	2.20E-16
	Gene body	1148 (38%)	0.549 ± 0.173	0.486 ± 0.168	2.20E-16
	TSS1500	273 (9%)	0.479 ± 0.215	0.469 ± 0.172	ns
	TSS200	113 (4%)	0.429 ± 0.248	0.426 ± 0.184	ns
	5′UTR	218 (7%)	0.481 ± 0.192	0.441 ± 0.171	0.02244
	3′UTR	105 (3%)	0.565 ± 0.193	0.529 ± 0.193	ns
	1st exon	54 (2%)	0.359 ± 0.238	0.381 ± 0.173	ns
	Intergenic region	1117 (37%)	0.521 ± 0.176	0.457 ± 0.159	2.20E-16
	CpG island	300 (10%)	0.432 ± 0.294	0.444 ± 0.204	ns
	CpG island shore	488 (16%)	0.5111 ± 0.215	0.478 ± 0.172	0.009
	CpG island shelves	316 (10%)	0.556 ± 0.175	0.495 ± 0.175	1.65E-05
	Open sea	1924 (64%)	0.531 ± 0.156	0.465 ± 0.160	2.20E-16
Low	All DMPs	1320	0.511 ± 0.340	0.497 ± 0.240	ns
	Gene body	431 (33%)	0.633 ± 0.302	0.580 ± 0.222	0.00395
	TSS1500	183 (14%)	0.379 ± 0.332	0.406 ± 0.229	ns
	TSS200	111 (8%)	0.277 ± 0.296	0.330 ± 0.196	ns
	5′UTR	85 (6%)	0.401 ± 0.341	0.433 ± 0.252	ns
	3′UTR	48 (4%)	0.666 ± 0.278	0.607 ± 0.213	ns
	1st exon	45 (3%)	0.247 ± 0.289	0.328 ± 0.208	ns
	Intergenic region	417 (32%)	0.537 ± 0.330	0.512 ± 0.229	ns
	CpG island	379 (29%)	0.346 ± 0.335	0.380 ± 0.228	ns
	CpG island shore	316 (24%)	0.478 ± 0.338	0.470 ± 0.236	ns
	CpG island shelves	130 (10%)	0.685 ± 0.269	0.626 ± 0.197	0.0441
	Open sea	495 (38%)	0.612 ± 0.303	0.569 ± 0.219	0.0124

*Average ± SD. TSS1500 and TSS200 mean the regions 1,500 and 200 bp upstream, respectively, of the transcription start site. CpG island shore indicates the region 2 kb away from the CpG island. CpG island shelf indicates the region 2 kb away from the CpG island shore. Open sea indicates the inter-CpG island region. DMP, differentially methylated probe; UTR, untranslated region; ns, not significant.

We then identified 616 DMPs (386 hypermethylated and 230 hypomethylated DMPs) shared by both haloperidol dose groups (Supplementary Table 1). None showed inverse changes between the two doses. GO analysis of the hypermethylated DMP-associated genes showed that the genes were mostly enriched in the transmembrane transporter complex- and ion channel-related GO terms (Supplementary Table 2). Interestingly, GO terms such as NMDA selective glutamate receptor complex, social behavior, memory and learning were also listed. On the other hand, the hypomethylated CpG-associated genes were enriched in GO terms such as nephric duct formation, respiratory system development, and lung development (Supplementary Table 3).

### Comprehensive DNA Methylation Analysis of Risperidone

We identified 476 and 1,025 DMPs in the high- and low-dose risperidone groups, respectively. Similar to haloperidol, DMPs in both risperidone groups showed hypermethylation and were mainly located much farther from the promoter regions, including the gene body, 3′ UTR, and intergenic region (Table 2). In total, over 60% of DMPs showed an increase in DNA methylation level, and the rate was increased to approximately 80% or more in the gene body and 3′-UTR at both doses (Supplementary Figure 2). In the context of CpG islands, significant differences were found in the open seas, and hypermethylated probes were enriched in the shelves and open seas at both doses (Table 2 and Supplementary Figure 2).

**TABLE 2:** Average β values of DMPs in the risperidone treatment group according to the genomic positions.

Dose	Context	# of probes	β value (risperidone)*	β value (control)*	*P* value
High	All DMPs	476 (100%)	0.558 ± 0.317	0.518 ± 0.224	0.0221
	Gene body	157 (33%)	0.674 ± 0.266	0.593 ± 0.195	0.002389
	TSS1500	62 (13%)	0.438 ± 0.333	0.429 ± 0.228	ns
	TSS200	40 (8%)	0.387 ± 0.323	0.385 ± 0.221	ns
	5′UTR	25 (5%)	0.541 ± 0.324	0.525 ± 0.229	ns
	3′UTR	15 (3%)	0.750 ± 0.118	0.621 ± 0.111	0.004727
	1st exon	16 (3%)	0.252 ± 0.260	0.314 ± 0.166	ns
	Intergenic region	161 (34%)	0.549 ± 0.317	0.519 ± 0.225	ns
	CpG island	130 (27%)	0.430 ± 0.340	0.425 ± 0.225	ns
	CpG island shore	116 (24%)	0.520 ± 0.324	0.480 ± 0.228	ns
	CpG island shelves	41 (9%)	0.673 ± 0.273	0.619 ± 0.199	ns
	Open sea	189 (40%)	0.646 ± 0.269	0.582 ± 0.197	0.009143
Low	All DMPs	1025 (100%)	0.538 ± 0.329	0.508 ± 0.228	0.016
	Gene body	330 (32%)	0.673 ± 0.269	0.599 ± 0.195	5.95E-05
	TSS1500	129 (13%)	0.387 ± 0.326	0.411 ± 0.221	ns
	TSS200	82 (8%)	0.320 ± 0.320	0.360 ± 0.214	ns
	5′UTR	68 (7%)	0.408 ± 0.331	0.417 ± 0.225	ns
	3′UTR	32 (3%)	0.718 ± 0.242	0.623 ± 0.177	ns
	1st exon	42 (4%)	0.227 ± 0.258	0.312 ± 0.175	ns
	Intergenic region	342 (33%)	0.564 ± 0.318	0.522 ± 0.222	0.04743
	CpG island	292 (28%)	0.362 ± 0.330	0.389 ± 0.217	ns
	CpG island shore	232 (23%)	0.504 ± 0.332	0.479 ± 0.229	ns
	CpG island shelves	107 (10%)	0.719 ± 0.245	0.639 ± 0.180	0.007554
	Open sea	394 (38%)	0.639 ± 0.281	0.577 ± 0.202	0.0004007

*Average ± SD. TSS1500 and TSS200 mean the regions 1,500 and 200 bp upstream, respectively, of the transcription start site. CpG island shore indicates the region 2 kb away from the CpG island. CpG island shelf indicates the region 2 kb away from the CpG island shore. Open sea indicates the inter-CpG island region. DMP, differentially methylated probe; UTR, untranslated region; ns, not significant.

We identified 377 DMPs shared by both doses of risperidone, which consisted of 254 hypermethylated and 123 hypomethylated DMPs (Supplementary Table 1). GO analysis of the hypermethylated DMP-associated genes showed enrichment in the regulation of neurotransmitter receptor activity, lipoprotein lipase activity, and ionotropic glutamate receptor complex (Supplementary Table 4). GO analysis of hypomethylated DMP-associated genes showed enrichment in dorsal/ventral pattern formation and odontogenesis of the dentin-containing tooth (Supplementary Table 5).

### Common Differentially Methylated Probes Between Haloperidol and Risperidone

**FIGURE 2:**
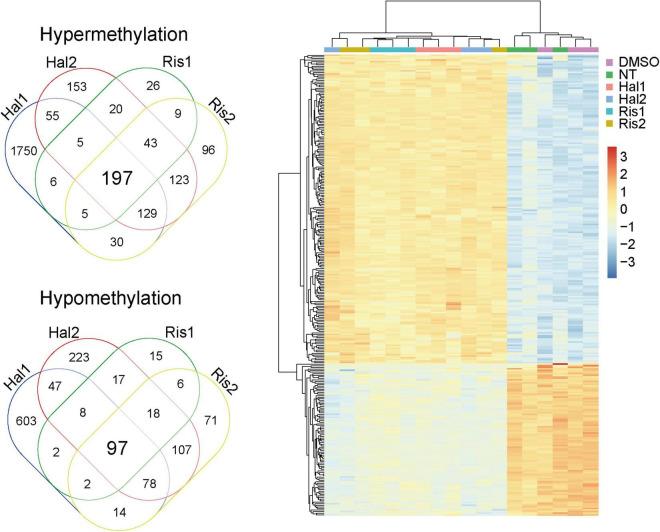
Venn diagram and cluster analysis of common DMPs. *Left*, Venn diagram of common DMPs between haloperidol and risperidone. *Right*, Two-way clustering of the 294 common DMPs. Data was centered mean to zero and scaled by dividing with standard deviations. DMP, differentially methylated probe.

**FIGURE 3:**
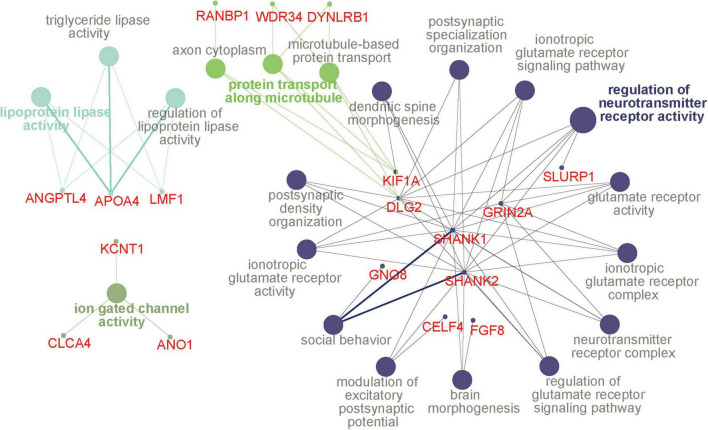
GO terms and hub genes. A picture was drawn based on the GO analysis of hypermethylated DMP-associated genes using ClueGO. Four functional groups were identified, and the color is the same for all pathways in a group. The circles with a bold font annotation represent the leading term (the most significant term of a group of pathways). Other terms are annotated in gray, and the smallest circles indicate genes related to the pathways. If the small circle is linked to two or more colors, it indicates that the gene is enriched in different pathways. The thickness of lines between pathways and genes is based on the GO evidence code. All experimental evidence codes, such as Inferred from Experiment (EXP) and Inferred from Direct Assay (IDA), have a thick edge, and others, such as Inferred from Electronic Annotation (ICA) and Inferred from Reviewed Computational Analysis (RCA), have a thin edge. DMP, differentially methylated probe; GO, gene ontology.

We then examined the common DMPs shared by haloperidol and risperidone. There were 294 common DMPs, consisting of 197 hypermethylated and 97 hypomethylated DMPs (Figure 2 and Supplementary Table 1). All DMPs showed DNA methylation changes in the same direction and were associated with a total of 189 genes. We listed the 13 DMPs showing robust DNA methylation changes (|Δβ| > 0.2) as representative (Table 3). GO analysis of the hypermethylated DMP-associated genes showed that genes related to the regulation of neurotransmitter receptor activity and lipoprotein lipase activity were enriched (Table 4). Interestingly, most of the neuronal function-related terms include *SHANK1* and *SHANK2* (Figure 3). In contrast, we did not find significant enrichment of GO terms in the hypomethylated DMP-associated genes.

**TABLE 3:** Common DMPs showing robust DNA methylation changes.

Probe	Gene	Genomic context	CpG island	Hal1		Hal2		Ris1		Ris2	
				
				Δβ	*P*	Δβ	*P*	Δβ	*P*	Δβ	*P*
cg17030628	IL20	TSS200	Open sea	0.92	2.48E-12	0.93	7.61E-11	0.94	1.59E-12	0.91	1.32E-11
cg14333542		Intergenic region	Open sea	−0.79	9.61E-11	−0.82	3.73E-11	−0.82	7.05E-11	−0.81	6.76E-11
cg04124606		Intergenic region	Open sea	−0.48	5.32E-10	−0.48	5.96E-10	−0.48	5.48E-10	−0.48	5.37E-10
cg19605788	CNTD2	Gene body	CpG island	0.37	1.83E-09	0.36	2.39E-09	0.37	1.89E-09	0.37	1.89E-09
cg20168823	BRUNOL4	Gene body	CpG island	0.36	2.98E-09	0.36	2.15E-09	0.36	1.96E-09	0.36	2.85E-09
cg17301379		Intergenic region	CpG island	0.40	1.42E-08	0.39	9.24E-09	0.40	7.84E-09	0.40	1.59E-08
cg21093807	C3orf63	TSS1500	CpG island	0.27	7.66E-08	0.28	4.57E-07	0.26	1.09E-06	0.26	2.16E-07
cg08052546		Intergenic region	CpG island shore	0.27	1.03E-07	0.22	5.31E-07	0.24	1.62E-07	0.23	1.34E-07
cg05545910	TTLL1	Gene body	Open sea	0.28	3.17E-06	0.31	1.05E-07	0.27	4.24E-08	0.29	5.11E-05
cg10442913	ATP1A1	TSS1500	CpG island	0.22	7.87E-06	0.23	7.65E-06	0.24	6.21E-06	0.22	7.89E-06
cg07369507	ZNF323	TSS1500	Open sea	−0.27	3.76E-05	−0.31	2.55E-05	−0.24	3.80E-06	−0.29	1.71E-07
cg21581312	LOC723972	TSS200	Open sea	0.31	6.19E-05	0.41	2.33E-05	0.32	3.58E-06	0.34	5.52E-06
cg11057824	C14orf182	Gene body	CpG island shore	0.23	0.000282	0.27	1.04E-05	0.25	2.12E-05	0.29	6.04E-06

*Hal1, high dose haloperidol group; Hal2, low dose haloperidol group; Ris1: high dose risperidone group, Ris2: low dose risperidone group. Δβ indicates average β (antipsychotics) – average β (control). TSS1500 and TSS200 mean the regions 1,500 and 200 bp upstream, respectively, of the transcription start site. CpG island shore indicates the region 2 kb away from the CpG island. Open sea indicates the inter-CpG island region. DMP, differentially methylated probe.*

**TABLE 4:** GO analysis of hypermethylated DMP-associated genes altered by both haloperidol and risperidone treatment.

GO ID	GO term	Category	Corrected *P* value	# of genes	Gene
GO:0099601	Regulation of neurotransmitter receptor activity	BP	0.00345	5	DLG2, GRIN2A, SHANK1, SHANK2, SLURP1
GO:0004465	Lipoprotein lipase activity	MF	0.00585	3	ANGPTL4, APOA4, LMF1
GO:0051004	Regulation of lipoprotein lipase activity	BP	0.00586	3	ANGPTL4, APOA4, LMF1
GO:0008328	Ionotropic glutamate receptor complex	CC	0.00586	4	DLG2, GRIN2A, SHANK1, SHANK2
GO:0098878	Neurotransmitter receptor complex	CC	0.00594	4	DLG2, GRIN2A, SHANK1, SHANK2
GO:0060997	Dendritic spine morphogenesis	BP	0.0071	3	KIF1A, SHANK1, SHANK2
GO:0098815	Modulation of excitatory postsynaptic potential	BP	0.01065	3	CELF4, SHANK1, SHANK2
GO:0099118	Microtubule-based protein transport	BP	0.01086	4	DLG2, DYNLRB1, KIF1A, WDR34
GO:0098840	Protein transport along microtubule	BP	0.01086	4	DLG2, DYNLRB1, KIF1A, WDR34
GO:1904115	Axon cytoplasm	CC	0.01098	3	DLG2, KIF1A, RANBP1
GO:0048854	Brain morphogenesis	BP	0.01101	3	FGF8, SHANK1, SHANK2
GO:1900449	Regulation of glutamate receptor signaling pathway	BP	0.0111	4	DLG2, GRIN2A, SHANK1, SHANK2
GO:0097106	Postsynaptic density organization	BP	0.0117	3	DLG2, SHANK1, SHANK2
GO:0008066	Glutamate receptor activity	MF	0.01179	4	DLG2, GRIN2A, SHANK1, SHANK2
GO:0099084	Postsynaptic specialization organization	BP	0.01218	3	DLG2, SHANK1, SHANK2
GO:0004970	Ionotropic glutamate receptor activity	BP	0.01224	4	DLG2, GRIN2A, SHANK1, SHANK2
GO:0022839	Ion gated channel activity	BP	0.01253	3	ANO1, CLCA4, KCNT1
GO:0004806	Triglyceride lipase activity	MF	0.01327	3	ANGPTL4, APOA4, LMF1
GO:0035235	Ionotropic glutamate receptor signaling pathway	BP	0.01349	4	DLG2, GRIN2A, SHANK1, SHANK2
GO:0035176	Social behavior	BP	0.01574	3	GNG8, SHANK1, SHANK2

*GO, gene ontology; DMP, differentially methylated probe; BP, biological process; MF, molecular function; CC, cellular component.*

### DNA Methylation Changes of Pharmacological Targets of Antipsychotics

We then examined the effects of haloperidol and risperidone on DNA methylation of the pharmacological targets of antipsychotics, including *DRD2* and *HTR2A*. In *DRD2*, a probe (cg21013206) showed a significant increase in DNA methylation (Δβ = 0.102, *P* = 0.006) in the high-dose haloperidol group. This probe is located near the *DRD2* promoter, suggesting a possible suppressing effect on its gene expression. In *HTR2A*, a probe (cg15894389) showed a significant decrease in DNA methylation (Δβ = −0.110, *P* = 0.0109) in the low-dose risperidone group. This probe is located within the first exon.

## Discussion

We comprehensively examined DNA methylation changes induced by a typical antipsychotic, haloperidol, and a second-generation atypical antipsychotic, risperidone. To the best of our knowledge, this is the first study to examine the common effect of two distinctive antipsychotics on DNA methylation in human neuroblastoma cells. Haloperidol acts as a DRD2 antagonist to ameliorate positive symptoms. Risperidone antagonizes HTR2A as well as DRD2, thus improving both positive and negative symptoms. Our study found that haloperidol could induce DNA methylation changes at more sites than risperidone, which may be related to its stronger dopamine antagonism. In addition, our study identified the co-action sites of these two antipsychotics, both associated with regulation of neurotransmitter receptor activity and glutamate receptor activity. These results indicate that the epigenetic effects of the two antipsychotics have their own characteristics and some similarities, which are worthy of further study in the future.

The hypermethylated CpG sites in the high-dose haloperidol group were mainly located in the regions away from the promoters and the CpG island. Similarly, they were mainly located in the gene body in the low-dose group. Although risperidone induced fewer DNA methylation changes than haloperidol, risperidone also induced hypermethylation at CpG sites located away from the promoter and CpG island. These patterns of changes were closely similar to those with blonanserin (Murata et al., 2014) and perospirone (Murata et al., 2019) using the same cell culture model and suggest that antipsychotics generally increase DNA methylation in a dose-dependent manner and that genomic regions less important for gene expression are more prone to be altered by antipsychotics. This was also concordant with a previous study that revealed an association of haloperidol and increased global DNA methylation levels in leukocytes (Melas et al., 2012). It should be noted that the possible role of intergenic DNA methylation is to repress the activity of harmful genetic elements such as transposons (Pappalardo and Barra, 2021). DNA methylation of the gene body is also associated with altered gene expressions (Moore et al., 2013). Therefore, DNA methylation changes at these sites are also important.

Despite the different classes, these two antipsychotics showed very similar DNA methylation changes, especially at low doses, as evidenced by cluster analysis and PCA (Figure 1). We identified 294 common DNA methylation changes between the two antipsychotics regardless of the dose. They showed the same direction of changes in DNA methylation, which mainly consisted of hypermethylation (Figure 2). The GO and pathway analyses revealed that the hypermethylated genes were included in the GO terms related to neuronal functions such as regulation of neurotransmitter receptor activity and ionotropic glutamate receptor complex (Table 4). We identified two key DMPs, cg02631082 and cg12198334, related to *SHANK1* and *SHANK2*, respectively, that were involved in these GO terms. Additionally, we observed that two probes, cg08447324 (Δβ = 0.117, *P* = 7.212e-05) and cg16658931 (Δβ = 0.114, *P* = 7.286e-05) in *SHANK1*, and two probes (cg10911054 (Δβ = 0.132, *P* = 2.207e-06) and cg18080819 (Δβ = 0.126, *P* = 1.984e-04) in *SHANK2* also showed hypermethylation in the high-dose haloperidol group. *SHANK* genes encode scaffold proteins in postsynaptic neurons and are involved in neuropsychiatric disorders (Kursula, 2019; Taylor et al., 2020). Epigenetic regulation of *SHANK* genes by antipsychotics affects a broad range of neuronal functions.

Among the genes showing robust DNA methylation changes, *IL20* showed the largest changes. IL20 is a cytokine belonging to the IL10 family, which also includes several other interleukins. In the central nervous system, these cytokines are upregulated or downregulated in response to environmental insults in glial cells and are involved in neuroinflammation (Burmeister and Marriott, 2018). In contrast to the well-characterized neuroprotective role of IL10, the role of IL20 remains unclear and is considered to play a proinflammatory factor in some cases (Burmeister and Marriott, 2018). In schizophrenia, increased proinflammatory cytokines may be involved in its etiology (Lesh et al., 2018; Sahbaz et al., 2020). Interestingly, haloperidol and risperidone showed opposite effects on the induction of cytokines in astroglial cell lines (Bobermin et al., 2018). Haloperidol induced the production of anti-inflammatory factors such as IL10, whereas risperidone decreased the production of these factors. Although the expression level of *IL20* was not previously examined, our epigenetic study suggests that they have common suppressive effects on *IL20*.

Two of the other top DMP-associated genes, *ATP1A1* and *ZNF323*, were also involved in psychiatric disorders and neural function. *ATP1A1* encodes the a1 subunit of the ubiquitously expressed ouabain-sensitive Na+, K+-ATPase pump. Mutations in this gene are involved in Charcot-Marie-Tooth disease (Lassuthova et al., 2018). One study showed its downregulation in the brains of mice treated with lithium (Chetcuti et al., 2008). Both haloperidol and risperidone induced hypermethylation, suggesting a similar suppressive role in the expression of this gene. *ZNF323*, also known as *ZSCAN31*, encodes a multiple C2H2-type zinc finger domain-containing protein that plays an active role in embryonic development (Pi et al., 2002). Significant downregulation of *ZNF323* in the frontal cortex and hippocampus of schizophrenia patients has been reported (Luo et al., 2015). The probe associated with *ZNF323* was hypomethylated by these antipsychotics.

Among the probes related to *DRD2*, one probe (cg21013206) reported in blonanserin-treated cells (Murata et al., 2014) overlapped in the high-dose haloperidol group. In addition, two probes (cg22458194 and cg03691958) in the coding region of *DRD2*, reported in perospirone-treated cells (Murata et al., 2019), also showed DNA methylation changes in the risperidone group, but the changes did not reach the significance level (data not shown). Interestingly, the changes at these probes usually represent increased DNA methylation, suggesting the suppressive effect of *DRD2* gene expression. DNA methylation in the important CpG sites for *DRD2* expression may modulate the effect of antipsychotics by repressing its gene expression. The incidence of extrapyramidal symptoms (EPS) in haloperidol is higher than that in risperidone, which is related to the strong DRD2 antagonism of haloperidol. Our study suggested that haloperidol can repress the expression of *DRD2* through its hypermethylation, which may provide some evidence to explain the occurrence of EPS in haloperidol from the epigenetic perspective.

From the comparison with previous studies, *CACNA1A* showed the highest DNA methylation change by blonanserin in the same cell culture model (Murata et al., 2014). In *CACNA1A*, one probe (cg26554567) showed a significant decrease in DNA methylation in the low-dose haloperidol group (Δβ = −0.109, *P* = 1.382e-05) and the high-dose risperidone group (Δβ = −0.107, *P* = 4.339e-05). *GRM7* has been reported to be a potential target gene for seven antipsychotics, including haloperidol and risperidone (Liang et al., 2020). In this study, one probe (cg21032008) related to *GRM7* showed a significant increase in DNA methylation (Δβ = 0.117, *P* = 0.0069) in the high-dose haloperidol group compared to the control group.

In this study, we used a human neuroblastoma cell line for systematic comparison of the epigenetic effects of antipsychotics. Therefore, the DNA methylation changes identified here might be quite different from those identified in postmitotic neuronal cells. Although the SK-N-SH expresses *DRD2* and *HTR2A* very weakly at the transcript level, according to the DepMap database,^4^ it is not clear if DNA methylation changes identified in this study were caused by antagonism of these receptors. In addition, the molecular mechanism underlying DNA methylation changes remains unclear. Among the ingredients in the medium, FBS contains many uncharacterized chemicals. This may induce the DNA methylation changes during culture, independent of antipsychotics. However, we put the two control groups (no antipsychotics and DMSO only) and applied the different concentrations of antipsychotics. Therefore, we minimized the effect of the culture medium. In addition, it is not clear whether DNA methylation changes contribute to pharmacological efficacy. Therefore, future studies using primary cultured cells and/or animal models of psychiatric disorders will be required to clarify the true effects.

## Data Availability Statement

The datasets presented in this study can be found in online repositories. The names of the repository/repositories and accession number(s) can be found below: https://www.ncbi.nlm.nih.gov/search/all/?term=GSE185973.

## Author Contributions

MB performed the experiments. YN performed the data management. JD, YN, and TK analyzed the data. JD, YN, SF, MB, and KI wrote the manuscript. KK, MB, and KI designed the experiments. All authors discussed the results and commented on the manuscript.

## Conflict of Interest

The authors declare that the research was conducted in the absence of any commercial or financial relationships that could be construed as a potential conflict of interest.

## Publisher’s Note

All claims expressed in this article are solely those of the authors and do not necessarily represent those of their affiliated organizations, or those of the publisher, the editors and the reviewers. Any product that may be evaluated in this article, or claim that may be made by its manufacturer, is not guaranteed or endorsed by the publisher.

## Footnotes

1
http://www.r-project.org/

2
http://www.sthda.com/english/rpkgs/factoextra

3
http://bioinformatics.psb.ugent.be/webtools/Venn/

4
https://doi.org/10.6084/m9.figshare.15160110.v2
