# Supplementary material for: Comprehensive DNA Methylation Analysis of Human Neuroblastoma Cells Treated With Haloperidol and Risperidone
Source: Front Mol Neurosci. 2021 Dec 6;14:792874. doi: 10.3389/fnmol.2021.792874 (PMC8687450; doi:10.3389/fnmol.2021.792874)
Supplement: Supplementary file 1 [file Image_1.pdf]

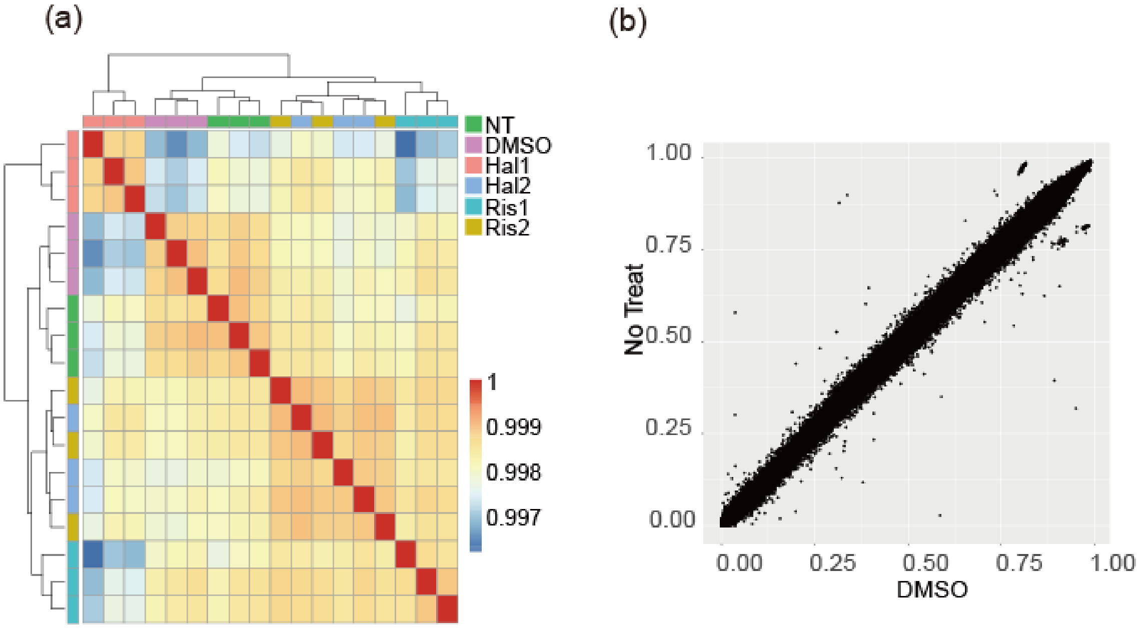

Supplementary Figure S1. Heatmap and scatter plot of samples. (a) Heatmap cluster of all the samples. NT, no treatment group; DMSO, dimethyl sulfoxide group; Hal1: haloperidol high dose group; Hal2: haloperidol low dose group; Ris1: risperidone high dose group; Ris2: risperidone low dose group. (b) Scatter plot of the NT and DMSO groups. Average β values were compared.

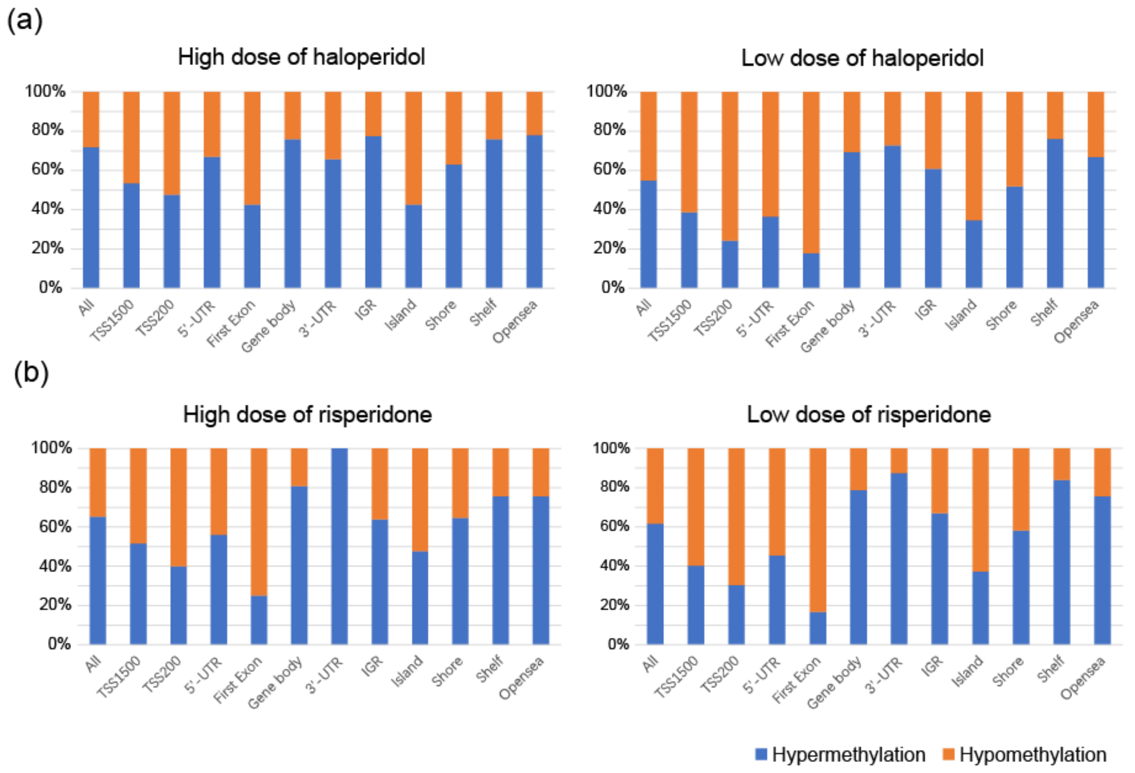

Supplementary Figure S2. Proportion of hypermethylated and hypomethylated DMPs in the haloperidol (a) and risperidone (b) groups. TSS1500 and TSS200 indicate the regions 1500 bp and 200 bp upstream, respectively, of the transcription start site. IGR indicates the intergenic region. Shore indicates the region 2 kb away from the CpG island. Shelf indicates the region 2 kb away from shore. Open sea indicates the inter-CpG island region. DMP, differentially methylated probe; UTR, untranslated region.
